# Multimodal Validation of an sEMG-Based Visual Biofeedback System for Deep Abdominal Muscle Activation in Healthy Adults: A Randomized Controlled Proof-of-Concept Trial

**DOI:** 10.3390/healthcare14121606

**Published:** 2026-06-08

**Authors:** Hyewon Jeon, Sunbeom Park, Sungwoo Bang, Kisik Tae, Hyunju Lee

**Affiliations:** 1 Department of Physical Therapy, Konyang University, Daejeon 35365, Republic of Korea; 2 Department of Biomedical Engineering, Konyang University, Daejeon 35365, Republic of Korea

**Keywords:** transversus abdominis, electromyography, visual biofeedback, rehabilitative ultrasound imaging, motor control, core stabilization

## Abstract

Background: Portable biofeedback technologies are increasingly used in rehabilitation; however, the validity of surface electromyography (sEMG) as a surrogate indicator of deep abdominal muscle function remains unclear. This study aimed to validate a portable sEMG-based visual biofeedback system by examining its relationship with ultrasound-derived measures of deep abdominal muscle activation. Methods: Twenty-nine healthy adults were randomly assigned to a Visual Biofeedback group (n = 14) or a Verbal Feedback group (n = 15). Both groups performed a standardized 2-week core stabilization program. Muscle activation of the deep abdominal muscle complex (transversus abdominis–internal oblique; TrA–IO) and external oblique (EO) was measured using sEMG (%MVIC), while ultrasound imaging was used to assess transversus abdominis thickness and contractile activity (ADIM–Rest index). Between-group differences and correlations between EMG and ultrasound variables were analyzed. Results: The Visual Biofeedback group demonstrated significantly greater improvements in TrA–IO activation and in the preferential activation ratio (TrA–IO/EO) compared to the Verbal group (*p* = 0.004). Ultrasound analysis revealed significantly greater increases in TrA thickness and contractile activity in the Visual group (*p* < 0.001). A significant positive correlation was observed between changes in TrA–IO activation and TrA thickness (ρ = 0.51, *p* < 0.001). Conclusions: Portable sEMG-based visual biofeedback demonstrated physiological relevance by reflecting ultrasound-derived changes in deep abdominal muscle function. These findings support the use of sEMG as a practical surrogate tool for monitoring deep core muscle activation and highlight the potential of portable biofeedback systems in scalable and accessible rehabilitation.

## 1. Introduction

The transversus abdominis (TrA) plays a critical role in trunk stability through its feedforward activation prior to limb movement, contributing to spinal control and postural regulation [1,2]. Impaired activation and delayed recruitment of the TrA have been consistently reported in individuals with low back pain, highlighting the importance of selectively training the deep abdominal musculature in rehabilitation settings. The abdominal drawing-in maneuver (ADIM) is widely used to facilitate preferential activation of the TrA. However, due to the deep anatomical location of the muscle, it is difficult for individuals to accurately perceive and control its contraction. Conventional approaches relying on verbal and tactile feedback are often insufficient to ensure consistent and selective activation of the deep abdominal muscles [3,4].

To address these limitations, visual biofeedback techniques have been introduced to provide real-time information on muscle activity. Among these, rehabilitative ultrasound imaging is considered a gold-standard method for assessing and training deep abdominal muscle function, as it allows direct visualization of muscle thickness and contraction patterns [5,6,7]. However, despite its high validity, ultrasound-based biofeedback is limited in routine clinical practice due to its high cost, lack of portability, and requirement for skilled operators.

Recently, there has been increasing interest in portable and scalable digital health solutions that can be applied in both clinical and home-based rehabilitation settings. Surface electromyography (sEMG)-based biofeedback systems offer a cost-effective and accessible alternative, enabling real-time monitoring of muscle activity without the need for complex imaging equipment. However, due to the anatomical depth of the TrA and potential signal cross-talk from adjacent muscles, the validity of sEMG as a surrogate indicator of deep abdominal muscle activation remains unclear [8,9].

Therefore, establishing the physiological relevance of sEMG signals in reflecting deep abdominal muscle function is a critical step toward the broader clinical adoption of portable biofeedback systems.

The primary purpose of this study was to validate a portable sEMG-based visual biofeedback system by examining its ability to reflect changes in deep abdominal muscle activation, using ultrasound imaging as a reference standard. To achieve this, we compared the effects of sEMG-based visual biofeedback and conventional verbal feedback on neuromuscular activation and morphological changes in the deep abdominal muscle complex (TrA–IO) in healthy adults.

We hypothesized that the sEMG-based visual biofeedback system would facilitate greater preferential activation of the deep abdominal muscles and demonstrate a significant association with ultrasound-derived measures, supporting its use as a practical surrogate tool for monitoring deep core muscle function in rehabilitation settings.

## 2. Methods

### 2.1. Study Design

**Figure 1:**
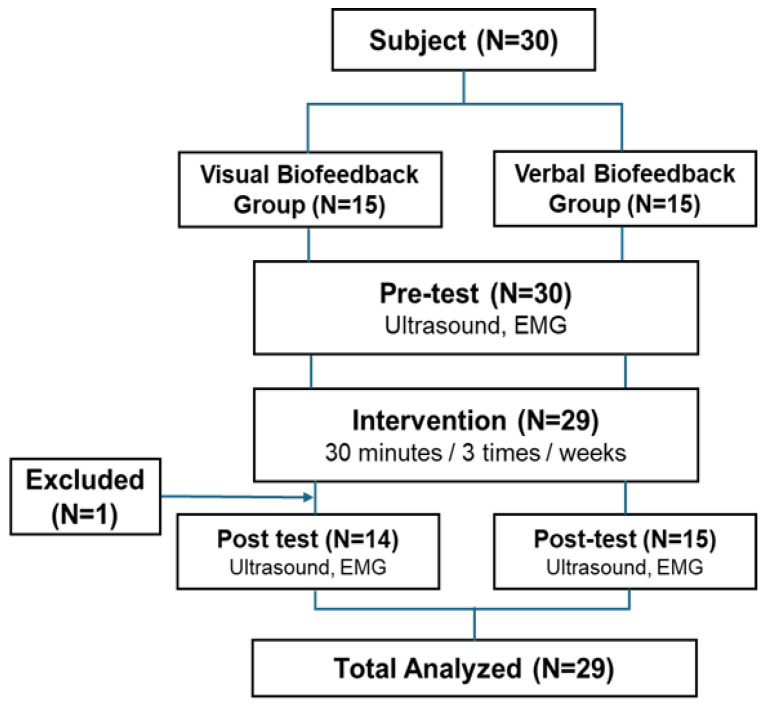
Flow chart.

This study was conducted as a randomized controlled trial (RCT) to compare the effects of visual biofeedback training and verbal feedback training on changes in the muscle activation and thickness of the transversus abdominis (TrA) over a two-week period. Participants were randomly assigned to either the visual biofeedback group (Group A) or the verbal feedback group (Group B). The participants were screened to ensure they were free of any musculoskeletal or neurological pathologies. However, prior experience with core-specific exercise programs (e.g., Pilates or Yoga) or their baseline physical activity levels were not utilized as specific exclusion criteria during the recruitment process. To minimize bias during the measurement and analysis processes, the assessor was blinded to the group assignments of the subjects. All subjects performed the same three exercises: the abdominal drawing-in maneuver (ADIM), side plank, and bridge exercise. The training was conducted three times a week for two weeks, totaling six sessions. Evaluation items were assessed before and after the intervention. The study procedure is illustrated in Figure 1.

### 2.2. Exercise Protocol

The exercises were conducted individually for each participant in a laboratory setting. Prior to the start of the intervention, all participants received 6 min of standardized education on how to perform the abdominal drawing-in maneuver (ADIM). The ADIM and bridge exercise were performed in the supine position, while the side plank was performed in the side-lying position. To minimize compensatory movements, participants were instructed to maintain a neutral pelvic position during all tasks. Each session consisted of the ADIM, side plank, and bridge exercise, in that order [10]. Each exercise involved maintaining an isometric contraction for 1 min followed by a 1 min rest. One set was defined as the completion of all three exercises, and a total of four sets were performed per session with a 2 min rest between sets. The exercise composition and duration were identical for both groups; only the type of feedback provided during the exercises differed according to the group assignment. To ensure that the intensity of instruction was comparable between the two groups, several control measures were implemented. First, both groups performed the exercises for the exact same duration, monitored by a digital timer, to prioritize the quality and sustainability of the contraction. Second, the control group received standardized verbal cues—specifically, "Maintain the feeling of drawing your navel toward your spine"—to minimize variability in the feedback provided. Consistent verbal encouragement was offered to both groups to maintain participant engagement and motivation throughout the session.

#### 2.2.1. Visual Biofeedback Training (Group A)

**Figure 2:**
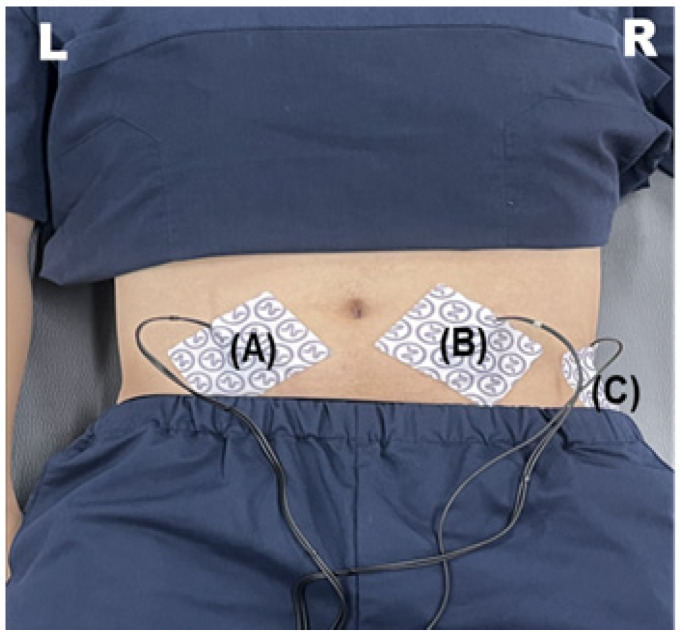
Visual feedback electrodes cable attachment region and direction. (A: Lt. Active electrode, B: Rt. Active electrode, C: Ground electrode; L: Left, R: Right).

Group A performed real-time surface electromyography (sEMG)-based visual biofeedback training using the Blue Back Physio (Red Balance, France). Electrodes were attached 2 cm medial and 2 cm inferior to the anterior superior iliac spine (ASIS) on both sides (Figure 2).

**Figure 3:**
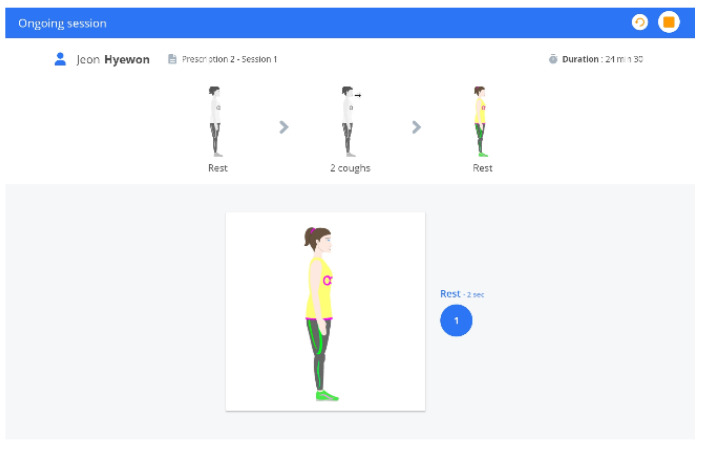
Calibration: Cough stage.

At the beginning of each session, an individualized signal was established through a calibration procedure within the device, which involved performing two standardized voluntary coughs. Coughing was selected as the reference contraction because it reflexively elicits near-maximal activation of the deep abdominal muscles (TrA and IO), providing a highly reproducible standard for normalization compared to voluntary isometric contractions which are often limited by motor control deficits [11]. Consequently, the peak EMG amplitude obtained during coughing was used to set the 100% reference threshold for the biofeedback training targets. Subsequently, participants performed the ADIM, side plank, and bridge exercise while self-regulating their deep abdominal muscle activation to ensure that the graph remained within the individualized target range displayed on the screen (Figure 3). In this study, the biofeedback signal from this electrode location was interpreted as deep abdominal muscle complex activity, corresponding to TrA–IO complex activity, rather than isolated TrA activity. This operational definition is based on the anatomical proximity and functional synergy of these muscles in the target region.

If the signal moved outside this range, participants were instructed to adjust their muscle contraction intensity to re-enter the target range. Each session lasted approximately 30 min.

#### 2.2.2. Verbal Feedback Training (Group B)

Group B performed the exercises receiving only verbal and tactile feedback provided by the researcher. During the exercises, the researcher provided standardized verbal instructions, such as “Gently pull your lower abdomen toward your spine, breathe comfortably, do not strain your upper abdomen, and keep your lower back and pelvis still”. For tactile feedback, subjects palpated the contraction of their abdominal muscles by placing their fingertips approximately 2 cm medial and inferior to the anterior superior iliac spine (ASIS) [12]. All subjects performed the abdominal drawing-in maneuver (ADIM), side plank, and bridge exercise using the same protocol, and the duration of each session was identical to that of Group A.

### 2.3. Participants

A total of 30 healthy volunteers aged 20–30 years were initially recruited for this study, and 29 participants were included in the final analysis. The sample size was calculated using G*Power 3.1.9.7 software (version 3.1.9.7; Heinrich Heine University, Düsseldorf, Germany). To determine the required number of participants, we assumed a large effect size (Cohen’s d = 1.0) based on previous studies that demonstrated the significant impact of visual biofeedback on transversus abdominis activation [12]. With a significance level (α) of 0.05 and a power (1-β) of 0.80 for a two-tailed independent *t*-test, the minimum required sample size was 17 participants per group (N = 34). Although we initially aimed for this number, a total of 29 participants completed the trial, and post hoc power analysis confirmed that this sample size was sufficient to detect significant differences in our primary outcomes.

All subjects were healthy adults with no history of low back pain or musculoskeletal diseases within the past six months. This study was approved by the Institutional Review Board (IRB) (Approval No.: 2025-05-023-001), and all participants provided written informed consent after receiving a thorough explanation of the study’s purpose and procedures.

The participants in this study consisted of adults aged 20–30 with a body mass index (BMI) ranging from 18.5 to 24.9. The inclusion criteria were limited to individuals capable of normally perceiving visual and auditory feedback who voluntarily agreed to participate after fully understanding the study’s objectives and procedures.

To ensure the integrity of the results and control for confounding variables, several exclusion criteria were applied. First, individuals with a history of surgery or trauma in the lumbar or abdominal region within the past six months were excluded. Furthermore, those with a current diagnosis of low back pain or musculoskeletal disorders—such as disc herniation or scoliosis—as well as individuals with neurological, cardiovascular, or respiratory diseases were also excluded. Finally, the study omitted participants who experienced difficulty in perceiving feedback or were currently taking medications that could potentially influence the research outcomes, such as muscle relaxants or analgesics.

### 2.4. Procedure

To recruit study participants, an announcement was posted on “E App,” an information-sharing platform used by subjects. Thirty men and women in their 20s and 30s recruited through the announcement underwent an initial screening survey to verify eligibility. After providing a thorough explanation of the research purpose and procedures to those meeting the inclusion criteria, written informed consent was obtained.

Prior to the intervention, participants’ general characteristics (sex, age, height, weight, and body mass index) were recorded on Case Report Forms (CRFs). Pre-intervention assessments were conducted as follows: the muscle thickness of the transversus abdominis (TrA), internal oblique (IO), and external oblique (EO) was measured using ultrasound imaging, and muscle activation was measured using surface electromyography (sEMG).

All participants participated in a total of six core exercise sessions over two weeks (three times per week). Each session lasted approximately 30 min and consisted of the abdominal drawing-in maneuver (ADIM), side plank, and bridge exercise. Based on random assignment, participants were divided into either the visual biofeedback group (Group A) or the verbal feedback group (Group B) and performed the same exercise protocol according to the feedback method defined for each group. Post-intervention assessments were performed using the same methods as the pre-assessments. All measurements were conducted by a pre-trained examiner. To minimize measurement error, both ultrasound and sEMG measurements were repeated three times, and the average values were used for analysis.

### 2.5. Experimental Protocol

#### 2.5.1. Surface Electromyography

**Figure 4:**
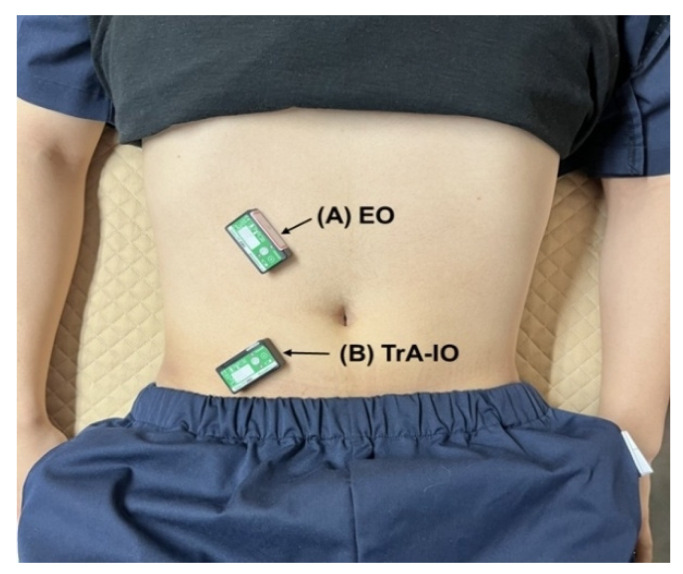
Electrode attachment region (A: External oblique (EO), B: transversus abdominis-internal oblique (TrA-IO)).

Surface electromyography (sEMG) was employed to quantify the muscle activation of the external oblique (EO) and the transversus abdominis internal oblique (TrA-IO) complex. Prior to electrode placement, the skin was shaved and disinfected with alcohol to minimize skin impedance [13]. Electrodes were oriented parallel to the muscle fibers: positioned just below the right 8th rib aiming inferomedially toward the pubis for the EO, and 2 cm medial and 2 cm inferior to the right anterior superior iliac spine (ASIS) for the TrA-IO channel (Figure 4) [8].

Due to the anatomical proximity and signal cross-talk between the transversus abdominis and internal oblique, the recorded sEMG signal was operationally defined as representing the deep abdominal muscle complex (TrA–IO), rather than an isolated measure of transversus abdominis activity.

Raw sEMG signals were acquired using a Biometrics DataLITE system at a sampling rate of 2000 Hz. To ensure signal integrity and eliminate movement artifacts, the raw data were conditioned using a fourth-order Butterworth band-pass filter with a frequency range of 20–450 Hz. Additionally, a 60 Hz notch filter was applied to remove electromagnetic power-line interference. Following full-wave rectification, the signals underwent smoothing via a root mean square (RMS) algorithm utilizing a 500 ms moving Triangle (Bartlett) window to accurately quantify muscle activation levels while mitigating crosstalk risks.

For amplitude normalization, maximal voluntary isometric contractions (MVIC) were established for each muscle. The EO MVIC was elicited via maximal trunk rotation against manual resistance away from the measured side, whereas the TrA-IO MVIC was obtained during maximal rotation toward the measured side. Each MVIC and the abdominal drawing-in maneuver (ADIM) were performed three times, with the average values utilized for analysis. The RMS values during the ADIM tasks were normalized to the average MVIC RMS and expressed as a percentage (%MVIC). Finally, the TrA-IO/EO ratio (%MVIC ratio) was calculated to specifically index the preferential neuromuscular recruitment of the deep abdominal complex relative to the superficial musculature. Given the anatomical proximity and potential signal cross-talk, the recorded sEMG signal was operationally defined as representing the deep abdominal muscle complex (TrA–IO), rather than a muscle-specific measure of transversus abdominis activity.

Due to the anatomical proximity of the TrA and IO, the recorded sEMG signals were operationally defined as activity of the TrA-IO complex rather than isolated TrA activity [14]. To verify the functional relevance of these signals, an internal cross-validation was performed using real-time B-mode ultrasound, which revealed a significant correlation between sEMG amplitude and morphological changes in TrA thickness (ρ = 0.51, *p* < 0.001). This suggests that the recorded signals meaningfully reflect deep abdominal muscle function.

#### 2.5.2. Ultrasound

The ultrasound assessments were performed according to a standardized protocol following 30 h of specialized training in abdominal imaging under expert supervision. The muscle thickness of the transversus abdominis (TrA), internal oblique (IO), and external oblique (EO) was evaluated using an ultrasound system (MySono U6, Samsung Medison, Seoul, Republic of Korea) equipped with a linear transducer (5–12 MHz). All measurements were performed at least two hours after a meal, with participants wearing comfortable clothing that allowed for abdominal exposure.

**Figure 5:**
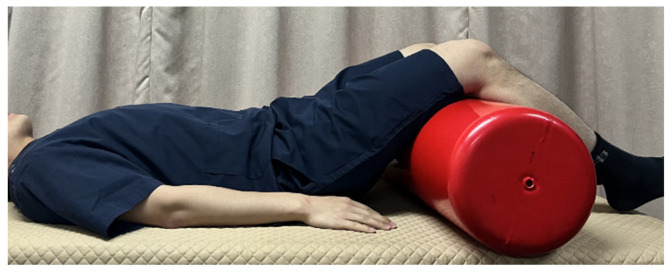
Crook-lying position for ultrasound imaging of the abdominal muscles.

**Figure 6:**
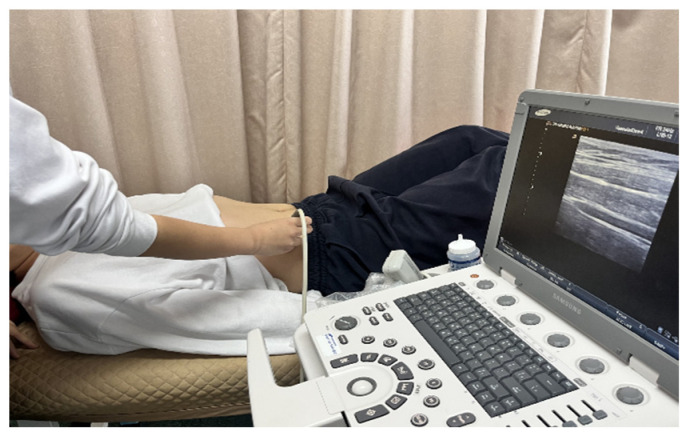
Ultrasound transducer placement over the lateral abdominal wall.

Participants were measured in the crook-lying position. A roll with a diameter of 25 cm was placed under the knees to maintain 60° of flexion in the hip and knee joints. The arms were placed straight beside the trunk, and the lower back was kept in a neutral position (Figure 5) [15]. The ultrasound transducer was positioned 2.5 cm medial to the mid-axillary line between the 12th rib and the iliac crest (Figure 6).

**Figure 7:**
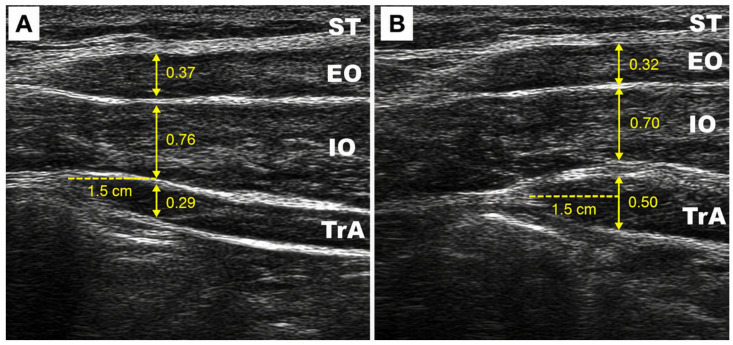
(**A**) Ultrasound image at rest; (**B**) ultrasound image during the abdominal drawing-in maneuver (ADIM). Muscle thickness was measured perpendicular to the fascia 1.5 cm from the TrA muscle–fascia junction.

Muscle thickness was determined by first identifying a point based on the TrA muscle–fascia junction in the ultrasound image and then measuring vertically at a distance of 1.5 cm from that point. The thickness of the TrA, IO, and EO was calculated respectively using this method, including only the muscle thickness while excluding the fascia (Figure 7) [15,16]. During the measurement, the transducer position was kept constant, and only the minimum pressure and angle adjustments necessary for image acquisition were allowed. Any instances where additional pressure was applied were recorded separately.

TrA lateral gliding was defined as the medial displacement of the anterior fascial tip of the TrA during ADIM. To ensure measurement consistency, ultrasound images were captured at rest and during contraction with the transducer maintained in a fixed position. The horizontal distance from the anterior fascial tip to the medial border of the ultrasound screen was measured in both states. Lateral gliding was then calculated as the difference between the resting and contraction distances [16,17].

Prior to the measurements, participants were instructed to familiarize themselves with the preferential TrA contraction method through pre-practice. All ultrasound images were captured at the end of expiration to minimize the effects of breathing. During measurements, the examiner provided basic breathing instructions such as “Breathe in” and “Breathe out,” along with a verbal cue for contraction: “Contract so that your navel moves toward your back”.

While one examiner operated the transducer, the other examiner saved the images according to the same signals [15]. All ultrasound evaluations were performed by the same examiner, and measurements were taken only on the right abdomen. To ensure the reliability of the measurements, intraclass correlation coefficients (ICCs) were calculated prior to the formal data collection. The intra-rater reliability for the two examiners showed excellent consistency, with ICC(1,1) values ranging from 0.92 to 0.96, confirming the technical proficiency of the assessors [18].

The thickness of each muscle was recorded as the average of three repeated measurements, with a 2 min rest provided between measurements to prevent muscle fatigue [19].

### 2.6. Data Analysis

All data collected in this study were analyzed using SPSS ver. 20.0 (IBM Corp., Armonk, NY, USA). The normality of the data distribution was assessed using the Shapiro–Wilk test. Since several variables—including EMG-derived changes (ΔTrA–IO, ΔEO, ΔTrA–IO/EO) and various ultrasound-derived measures (Rest, ADIM, and their differences)—did not satisfy the normality assumption, non-parametric tests were applied to maintain consistency across all analyses.

Accordingly, descriptive statistics are presented as medians and interquartile ranges (IQR). For within-group comparisons of pre- and post-intervention values, the Wilcoxon signed-rank test was used. To compare the intervention effects (calculated as Δ = Post − Pre) between the Visual Biofeedback and Verbal Feedback groups, the Mann–Whitney U test was employed. Additionally, an analysis of covariance (ANCOVA) was conducted as a supplementary analysis to verify between-group differences after adjusting for baseline values, with post-intervention values as the dependent variable and pre-intervention values as the covariate.

The relationship between changes in EMG activity and ultrasound-derived TrA thickness or gliding variables was examined using Spearman’s rank correlation coefficient. For all analyses, the statistical significance level was set at *p* < 0.05 (two-tailed). Effect sizes (*r*) for non-parametric tests were calculated as r = Z/√N and interpreted as small (0.1), medium (0.3), or large (0.5).

#### 2.6.1. EMG Analysis

Surface EMG was collected from the TrA–IO and EO muscles to quantify changes in abdominal muscle activity during the ADIM. Raw EMG signals were recorded by channel according to electrode placement and converted into time-domain indices through full-wave rectification and root mean square (RMS) processing during analysis. The resulting RMS values were normalized to the average RMS obtained during maximal voluntary isometric contraction (MVIC) and expressed as %MVIC.

After calculating the %MVIC for the TrA–IO and EO muscles for each subject, the TrA–IO/EO ratio (%MVIC ratio) was calculated to evaluate the relative activation of the TrA–IO channel. Consequently, three primary EMG indices were established: TrA–IO (%MVIC), EO (%MVIC), and the TrA–IO/EO ratio (%MVIC ratio). The intervention effect was defined as the pre-to-post difference in these indices (Δ = Post − Pre), denoted as ΔTrA–IO, ΔEO, and ΔTrA–IO/EO, respectively. Between-group comparisons for EMG change scores were performed using the Mann–Whitney U test, as normality was not met. Within-group comparisons of pre- and post-intervention EMG values were performed using the Wilcoxon signed-rank test. Because the EMG variables did not satisfy the normality assumption, the descriptive statistics for these variables were presented as median and interquartile range (IQR), rather than mean ± standard deviation. The r effect size was calculated using the Z-value from the Mann–Whitney U test and presented alongside the results.

#### 2.6.2. Ultrasound Analysis

Ultrasound image analysis targeted the lateral abdominal muscles: TrA, IO, and EO. Muscle thickness was quantified for each muscle at rest (Rest) and during the ADIM (ADIM) using consistent transducer placement and measurement criteria. The ADIM–Rest index (ADIM–Rest = ADIM thickness − Rest thickness) was calculated as the thickness difference at each time point (Pre, Post). This served as an ultrasound-based indicator of TrA contractile activity and abdominal muscle activation patterns [20,21].

Intervention-related changes were defined by calculating the difference (Δ = Post − Pre) for Rest, ADIM, and ADIM–Rest. Within-group changes in the ADIM–Rest index were evaluated using Wilcoxon signed-rank tests. Between-group comparisons for the change scores (Δ) of Rest, ADIM, and ADIM–Rest were performed using Mann–Whitney U tests following normality verification. Effect sizes were reported as r = Z/√N for non-parametric tests.

#### 2.6.3. Correlation Analysis Between Ultrasound and EMG

To quantitatively evaluate the physiological association between preferential TrA contraction and abdominal muscle activity, the correlation between the change in the ultrasound-based TrA preferential contraction index and the change in EMG-based activation was analyzed. Specifically, the relationship between the change in TrA ADIM–Rest preferential contraction (Δ TrA ADIM–Rest) and the change in TrA–IO channel activation (ΔTrA–IO %MVIC) was assessed using Spearman’s rank correlation analysis (Spearman’s ρ). Correlation magnitudes were interpreted as weak (ρ = 0.10–0.29), moderate (ρ = 0.30–0.49), or strong (ρ > 0.50), with the significance level set at *p* < 0.05 (two-tailed).

## 3. Results

### 3.1. Participant Characteristics

A total of 29 participants completed the study. One participant from the visual biofeedback group withdrew due to an unrelated injury. The final sample consisted of 14 participants in the Visual group and 15 in the Verbal group.

No significant differences were observed between groups in baseline characteristics, including age, sex distribution, height, and weight (all *p* > 0.05), confirming homogeneity between groups (Table 1).

### 3.2. Between-Group Differences in EMG Activity

Significant between-group differences were observed in deep abdominal muscle activation. The Visual group demonstrated a greater increase in TrA–IO activation (%MVIC) compared to the Verbal group (*p* = 0.004, r = 0.52).

Similarly, the preferential activation ratio (TrA–IO/EO) increased significantly more in the Visual group (*p* = 0.004, r = 0.53), indicating improved selective recruitment of the deep abdominal musculature, which is a key objective of ADIM-based training [3].

No significant between-group difference was observed in EO activation (*p* = 0.290, r = 0.20) (Table 2).

### 3.3. Within-Group Comparison of EMG Muscle Activity

In the Visual group, statistically significant changes were observed in both the TrA–IO and the external oblique (EO) (TrA–IO: *p* = 0.001; EO: *p* = 0.006). The effect sizes (r) were confirmed as 0.88 for the TrA–IO and 0.73 for the EO. In contrast, the Verbal group showed no statistically significant differences in any of the measured variables. Specifically, no significant differences were found for either the TrA–IO (*p* = 0.069, r = 0.47) or the EO (*p* = 0.100, r = 0.43) (Table 3).

**Figure 8:**
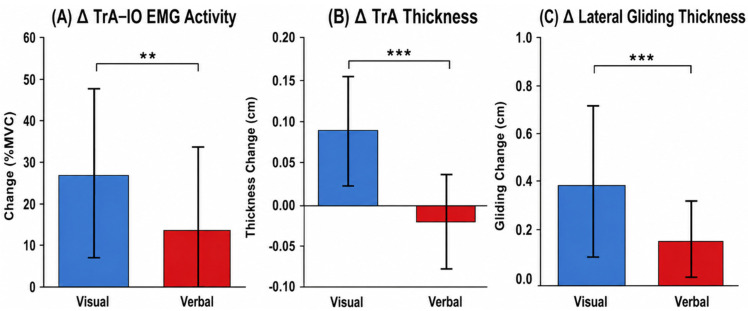
Comparison of therapeutic effects between the Visual Biofeedback and Verbal Feedback groups. (**A**) Changes in normalized TrA–IO EMG activity (%MVIC), showing superior recruitment in the Visual Biofeedback group. (**B**) Changes in TrA thickness (cm) measured by ultrasound during the Abdominal Drawing-in Maneuver (ADIM). (**C**) Comparison of TrA lateral gliding distances (cm). Data are presented as median change (Δ) with interquartile range (IQR) error bars. Significant differences between groups, determined by the Mann–Whitney U test, are indicated as ** *p* < 0.01, and *** *p* < 0.001.

The overall comparison of the delta (Δ) values clearly illustrates the superior training efficiency of the visual biofeedback. As shown in Figure 8, the Visual group achieved significantly greater improvements in the primary outcome metrics, including EMG activity, muscle thickness during ADIM, and gliding distance, compared to the Verbal group.

### 3.4. Between-Group Differences in Ultrasound Measures

Ultrasound analysis revealed significantly greater improvements in the Visual group for deep muscle function.

During ADIM, the Visual group showed a significantly greater increase in TrA thickness compared to the Verbal group (*p* < 0.001, r = 0.79). Similar patterns were observed for the internal oblique (IO) (*p* < 0.001, r = 0.64), while no significant difference was found for the external oblique (EO) (*p* = 0.505).

For the ADIM–Rest index, representing contractile activity, the Visual group demonstrated a significantly greater increase in TrA compared to the Verbal group (*p* < 0.001, r = 0.74). A significant difference was also observed for the IO (*p* = 0.001), whereas no difference was found for the EO (*p* = 0.331).

These findings are consistent with the use of ultrasound imaging as a valid method for assessing deep abdominal muscle contraction [5,21] (Table 4).

### 3.5. Changes in TrA Lateral Gliding

The Visual group demonstrated a greater increase in TrA lateral gliding compared to the Verbal group (*p* < 0.001, r = 0.77), suggesting improved dynamic interaction between abdominal muscle layers.

Given that fascial mobility and intermuscular coordination have been associated with functional movement patterns, these findings may reflect improved neuromuscular coordination (Table 5).

### 3.6. Correlation Between EMG and Ultrasound Measures

A significant positive correlation was observed between changes in TrA thickness (ADIM–Rest) and changes in TrA–IO EMG activity (ρ = 0.51, *p* < 0.001).

This finding is consistent with previous studies reporting an association between electromyographic activity and muscle thickness changes in abdominal muscles [7], supporting the physiological relevance of the sEMG measurements (Table 6).

**Figure 9:**
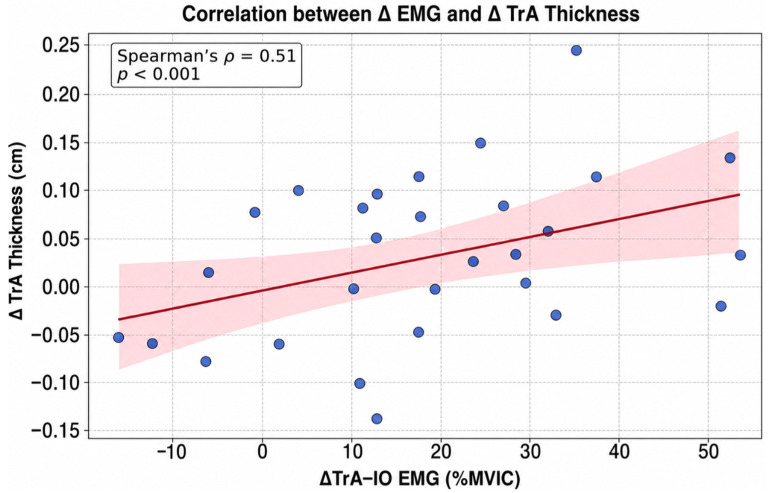
Correlation between EMG activity and morphological changes. Scatter plot illustrating the positive correlation between the change (Δ) in TrA − IO EMG activity and the change (Δ) in TrA thickness (Spearman’s ρ = 0.51, *p* < 0.001). The regression line (red) indicates that EMG-based feedback reflects structural muscle changes during ADIM.

The validity of the EMG was further confirmed by its correlation with structural changes. The scatter plot in Figure 9 demonstrates a strong positive correlation between Δ EMG activity and Δ TrA thickness (ρ = 0.51, *p* < 0.001), supporting the use of EMG as a reliable surrogate for monitoring deep abdominal muscle recruitment.

As a supplementary analysis, ANCOVA was performed to verify whether the main between-group differences remained significant after adjusting for baseline values. Significant group effects remained for post-intervention TrA thickness during ADIM, F(1,24) = 30.010, *p* < 0.001, and for post-intervention TrA–IO %MVIC, F(1,26) = 6.785, *p* = 0.015, after adjusting for the corresponding baseline values. These results were consistent with the primary change-score-based analyses.

Overall, the Visual biofeedback group demonstrated consistently greater improvements across key outcomes, including deep muscle activation (EMG), contractile thickness (ultrasound), and their association. These results indicate superior effectiveness of visual biofeedback in facilitating preferential activation of the deep abdominal muscle complex.

## 4. Discussion

The most important contribution of this study is not simply the demonstration of training effects, but the multimodal validation of a portable sEMG-based visual biofeedback system as a physiologically meaningful surrogate for deep abdominal muscle function. By integrating electromyographic data with ultrasound-derived morphological changes, this study provides evidence that portable sEMG signals reflect actual contractile behavior of the deep abdominal musculature rather than merely superficial muscle activation.

First, the present findings clearly demonstrated that visual biofeedback led to significantly greater improvements in TrA–IO activation and preferential activation ratio compared to verbal feedback. These results are consistent with previous studies showing that real-time feedback enhances selective activation of deep abdominal muscles and reduces compensatory activation of superficial muscles [3,12]. However, the current study extends these findings by demonstrating that these EMG-based improvements are accompanied by corresponding increases in ultrasound-derived TrA thickness and contractile activity, which strengthens the physiological interpretation of the EMG signals.

A particularly important finding is the significant correlation (ρ = 0.51) between changes in EMG activity and ultrasound-derived TrA contractile thickness. While previous studies have reported associations between EMG amplitude and muscle thickness [7], the present study provides validation within the context of a portable and clinically applicable biofeedback system. Given that ultrasound imaging is widely regarded as a valid method for assessing deep muscle function [5,20], this result supports the interpretation that sEMG—despite its limitations—can serve as a functionally meaningful surrogate marker when appropriately applied.

This validation aspect is critical because the clinical utility of ultrasound-based biofeedback is limited by cost, lack of portability, and operator dependency [5,6]. In contrast, portable sEMG systems offer a scalable alternative that can be used in routine clinical environments as well as home-based rehabilitation. Therefore, the present findings support a paradigm shift from gold-standard but impractical tools toward accessible surrogate technologies that maintain physiological relevance, which is highly aligned with current trends in digital and remote rehabilitation [22,23].

The superior performance observed in the visual biofeedback group can be explained by motor learning theory. Visual feedback promotes an external focus of attention, which has been shown to enhance motor efficiency and automaticity compared to internally focused verbal instructions [24,25]. In this study, real-time visual feedback likely enabled participants to continuously modulate their activation patterns, resulting in improved selective recruitment of the deep abdominal muscles while minimizing unnecessary activation of the external oblique. This interpretation is consistent with evidence that external focus enhances neuromuscular coordination and performance efficiency [24]. The superior performance in the visual biofeedback group can be further elucidated through the lens of Knowledge of Performance (KP). Unlike verbal instructions that often direct attention to internal body movements, the real-time sEMG visual display provided continuous KP, allowing participants to engage in active self-correction and self-organization of motor patterns. This process likely facilitated the mapping between augmented external feedback and the participants’ intrinsic proprioceptive feedback, potentially leading to more robust motor memory for deep abdominal recruitment compared to the transient effects of verbal cues. 

Furthermore, this process of motor learning suggests the potential for long-term retention of the acquired activation patterns even after the biofeedback device is removed. Previous evidence indicates that TrA training facilitated by visual cues can lead to sustained neuromuscular improvements that persist for several months beyond the initial training period [6,25,26].

Importantly, considering the short intervention duration of two weeks, the observed increases in TrA thickness and contractile indices are unlikely to reflect true hypertrophy. Instead, these changes should be interpreted as early-phase neuromuscular adaptations, including improved motor unit recruitment and intermuscular coordination [27,28,29]. The concurrent improvement in both EMG and ultrasound measures therefore reflects enhanced motor control rather than structural muscle remodeling.

In addition, the observed increase in TrA lateral gliding suggests improved interaction between abdominal muscle layers. Previous studies have linked fascial mobility to functional movement and pain conditions [30,31]. However, due to the lack of standardized interpretation frameworks, these findings should be cautiously interpreted as indicative of improved neuromuscular coordination rather than direct fascial adaptation.

Several limitations of this study should be acknowledged. First, due to anatomical constraints, surface EMG cannot entirely isolate transversus abdominis (TrA) activity and is susceptible to cross-talk, primarily from the internal oblique (IO) [8,32]. Therefore, the EMG signals in this study should be interpreted as representing the TrA–IO complex rather than a single muscle.

Second, the findings may have limited generalizability as the study was conducted on healthy individuals [1,33]. Clinical populations, such as those with chronic low back pain (CLBP), often exhibit impaired motor control and may adopt compensatory strategies. For clinical application, it is recommended to adjust feedback thresholds to individualized levels to prevent over-recruitment of superficial muscles and to integrate visual biofeedback with expert verbal guidance to ensure correct neuromuscular patterns.

Third, while the final sample size (n = 29) was slightly below the initial target (N = 34), post hoc power analysis confirmed sufficient power to detect significant differences in primary outcomes. To address the small sample size and non-normal distribution of certain variables, conservative non-parametric tests (Mann–Whitney U and Wilcoxon signed-rank tests) were consistently applied to ensure the robustness of the findings and minimize Type I errors. Nevertheless, results related to secondary outcomes, such as muscle thickness, should be considered preliminary.

Finally, the study focused on short-term (2-week) adaptations without assessing long-term retention or functional carry-over. Additionally, prior core exercise experience was not controlled, which may influence initial TrA recruitment.

Despite these limitations, this study provides a meaningful framework for validating portable biofeedback technologies by linking electrical activity with morphological changes. Our findings support sEMG-based systems as clinically feasible and physiologically grounded tools for monitoring deep abdominal muscle activation. Future research should extend this work to clinical populations, optimize signal processing techniques to improve sEMG specificity, and evaluate long-term outcomes in real-world rehabilitation settings through longitudinal designs.

## 5. Conclusions

This study demonstrated that a portable sEMG-based visual biofeedback system is capable of reflecting changes in deep abdominal muscle activation, as evidenced by its association with ultrasound-derived measures of transversus abdominis function. Participants who received visual biofeedback showed greater improvements in preferential activation of the deep abdominal muscle complex compared to those receiving conventional verbal feedback, indicating enhanced neuromuscular control.

Importantly, the observed correlation between EMG activity and ultrasound-based contractile changes supports the validity of sEMG as a practical surrogate indicator of deep abdominal muscle function. These findings suggest that portable sEMG-based biofeedback systems may serve as accessible and scalable tools for facilitating and monitoring deep core muscle training in both clinical and home-based rehabilitation settings. Further research is warranted to confirm these findings in clinical populations and to evaluate long-term outcomes.

## Figures and Tables

**Table 1:** General characteristics of subjects.

Variable	Visual (n = 14)	Verbal (n = 15)	*p*
Sex (male/female)	7/7	10/5	0.709
Age (years)	24.07 ± 4.22	22.60 ± 2.35	0.249
Height (cm)	168.93 ± 9.43	172.47 ± 9.09	0.305
Weight (kg)	63.27 ± 13.38	71.47 ± 11.38	0.081

Note. Values are presented as mean ± SD or n. *p*-values were obtained using independent *t*-tests (Age, Height, Weight) and chi-square test (Sex).

**Table 2:** Comparison of normalized EMG variables (Δ = Post − Pre) between Visual and Verbal Groups.

Muscle	Δ Visual	Δ Verbal	Test Statistic	*p*	Effect Size (r)
TrA–IO (%MVIC)	26.97 (38.66)	14.68 (35.86)	−2.79	0.004 **	0.52
EO (%MVIC)	7.12 (13.21)	3.30 (13.40)	−1.09	0.290	0.20
TrA–IO/EO (%MVIC ratio)	4.95 (18.06)	0.87 (2.86)	−2.85	0.004 **	0.53

Note. Values are expressed as median (interquartile range). Δ denotes the change from pre- to post-intervention (Post-Pre). Between-group comparisons were conducted using the Mann–Whitney U test. The effect size, r, was calculated as Z/√N and interpreted as small (0.1), medium (0.3), or large (0.5). ** *p* < 0.01.

**Table 3:** Within-Group Comparison of Normalized EMG Activity.

Group	Muscle	Pre (%MVIC)	Post (%MVIC)	Z	*p*	Effect Size (r)
Visual	TrA–IO	5.94 (11.59)	40.42 (47.99)	−3.30	0.001 **	0.88
	EO	6.60 (11.09)	18.62 (17.44)	−2.73	0.006 **	0.73
Verbal	TrA–IO	26.72 (30.12)	29.77 (42.55)	−1.82	0.069	0.47
	EO	13.05 (11.20)	17.36 (15.15)	−1.65	0.100	0.43

Note. Values are presented as median (interquartile range). EMG amplitudes are normalized to MVIC and expressed as %MVIC. Within-group comparisons (Pre vs. Post) were performed using the Wilcoxon signed-rank test. The effect size (r) was calculated as Z/√N. Significant differences are indicated as ** *p* < 0.01.

**Table 4:** Comparison of Muscle Thickness and Contraction Indices (Adim-Rest) between Visual and Verbal Groups (Δ = Post − Pre).

Condition	Muscle	Δ Visual	Δ Verbal	Z	*p*	Effect Size (r)
ADIM	TrA	0.14 (0.09)	−0.04 (0.14)	−4.28	<0.001 ***	0.79
	IO	0.16 (0.13)	−0.07 (0.25)	−3.45	<0.001 ***	0.64
	EO	0.00 (0.13)	0.02 (0.11)	−0.68	0.505	0.13
Rest	TrA	0.07 (0.07)	−0.01 (0.06)	−2.80	0.004 **	0.52
	IO	−0.02 (0.15)	−0.05 (0.21)	−0.74	0.477	0.14
	EO	0.03 (0.10)	0.06 (0.12)	−0.81	0.425	0.15
ADIM–Rest	TrA	0.09 (0.14)	−0.02 (0.12)	−4.00	<0.001 ***	0.74
	IO	0.14 (0.18)	−0.01 (0.15)	−3.08	0.001 **	0.57
	EO	−0.03 (0.13)	−0.17 (0.06)	−1.01	0.331	0.19

Note. Values are presented as median (interquartile range, IQR). Δ indicates the change from pre- to post-intervention (Post − Pre). For the ADIM–Rest contraction index, Δ was calculated as ΔADIM − ΔRest. Between-group comparisons were performed using the Mann–Whitney U test. Exact two-tailed *p*-values were reported. Effect size was reported as r, calculated as |Z|/√N. ** *p* < 0.01 and *** *p* < 0.001.

**Table 5:** Comparison of Gliding between Visual and Verbal groups (Δ).

Δ Visual	Δ Verbal	Z	*p*	Effect Size (r)
0.38 (0.61)	0.17 (0.26)	−4.14	<0.001 ***	0.77

Note. Values are presented as median (interquartile range, IQR). Δ indicates the change from pre- to post-intervention (Post − Pre). Between-group comparisons were performed using the Mann–Whitney U test. Effect size was reported as r, calculated as |Z|/√N. *** *p* < 0.001.

**Table 6:** Correlation between changes in ultrasound and EMG variables.

Variables	Spearman’s ρ	*p*
ΔTrA ADIM–Rest–ΔTrA/IO EMG (%MVIC)	0.51	<0.001 ***

Note. Values represent Spearman’s rank correlation coefficients (ρ). Δ indicates the change between pre- and post-intervention values. Correlations were interpreted as weak (0.10–0.29), moderate (0.30–0.49), and strong (≥0.50). *** *p* < 0.001.

## Data Availability

The datasets used and analyzed during the current study are available from the corresponding author upon reasonable request.
